# The emerging role of ferroptosis in intestinal disease

**DOI:** 10.1038/s41419-021-03559-1

**Published:** 2021-03-17

**Authors:** Shu Xu, Yao He, Lihui Lin, Peng Chen, Minhu Chen, Shenghong Zhang

**Affiliations:** Division of Gastroenterology, The First Affiliated Hospital, Sun Yat-sen University, Guangzhou, P. R. China

**Keywords:** Cell death, Intestinal diseases

## Abstract

Ferroptosis is a newly recognised type of regulated cell death (RCD) characterised by iron-dependent accumulation of lipid peroxidation. It is significantly distinct from other RCDs at the morphological, biochemical, and genetic levels. Recent reports have implicated ferroptosis in multiple diseases, including neurological disorders, kidney injury, liver diseases, and cancer. Ferroptotic cell death has also been associated with dysfunction of the intestinal epithelium, which contributes to several intestinal diseases. Research on ferroptosis may provide a new understanding of intestinal disease pathogenesis that benefits clinical treatment. In this review, we provide an overview of ferroptosis and its underlying mechanisms, then describe its emerging role in intestinal diseases, including intestinal ischaemia/reperfusion (I/R) injury, inflammatory bowel disease (IBD), and colorectal cancer (CRC).

## Facts

- Ferroptosis is a unique type of regulated cell death that involves iron accumulation and lipid oxidation.
- Ferroptosis has been linked to several diseases and cancers, but its role in intestinal disease is uncharacterised.
- Ferroptosis can be a positive and negative regulator of the disease.

## Open questions

- Does ferroptosis play a role in distinct forms of intestinal diseases?
- What contributes to ferroptosis in the occurrence and development of intestinal diseases? Are there unknown mechanisms and signalling pathways?
- Will ferroptosis-related factors be indicators of disease severity?

## Introduction

**Fig. 1:**
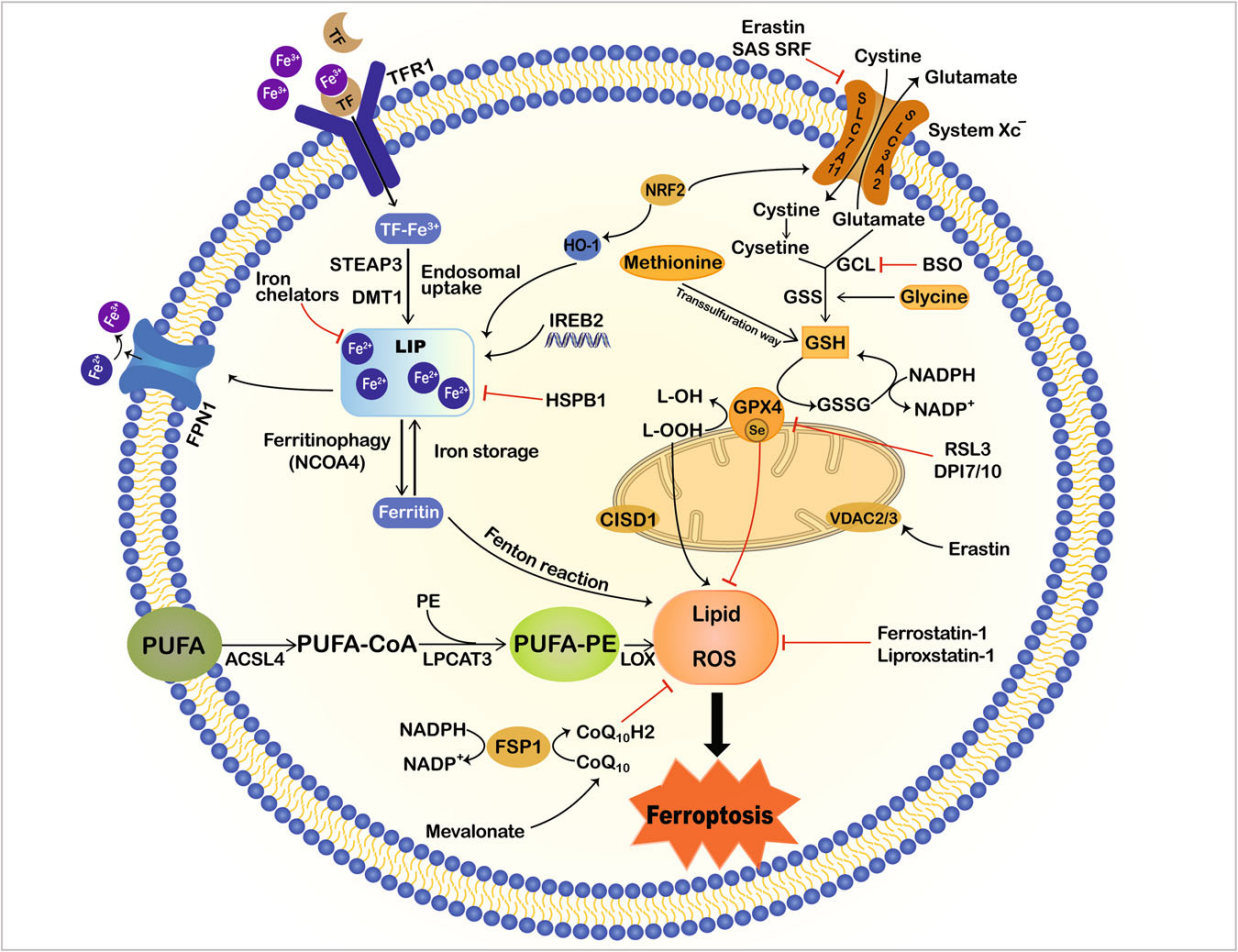
Mechanisms of ferroptosis. Ferroptosis is characterised by iron accumulation, excessive ROS production and overwhelming lipid peroxidation. Three main metabolic pathways, amino-acid/GSH, lipid, and iron pathways, participate in the initiation and execution of ferroptosis. Moreover, there are additional signalling pathways and regulators controlling ferroptosis sensitivity. This illustration shows the process of ferroptosis, summarising the key molecules and targets that regulate iron and lipid peroxidation. ACSL4 acyl-CoA synthetase long-chain family member 4, BSO buthionine sulphoximine, CISD1 CDGSH iron sulphur domain 1, DMT1 divalent metal transporter 1, FSP1 ferroptosis suppressor protein 1, FPN1 ferroportin 1, GPX4 glutathione peroxidase 4, GSH glutathione, GSSG oxidized glutathione, GSS glutathione synthetase, GCL glutamate-cysteine ligase, HO-1 haem oxygenase 1, HSPB1 heat shock protein beta-1, IREB2 iron response element-binding protein 2, LOX lipoxygenase, LPCAT3 lysophosphatidylcholine acyltransferase 3, NCOA4 nuclear receptor coactivator 4, NRF2 nuclear factor E2-related factor 2, PUFA polyunsaturated fatty acid, PE phosphatidylethanolamine, ROS reactive oxygen species, RSL3 Ras-selective lethal 3, STEAP3 six-transmembrane epithelial antigen of prostate 3 metalloreductase, SLC7A11 solute carrier family 7 member 11, SAS sulphasalazine, SRF sorafenib, TF transferrin, TFR1 transferrin receptor 1, VDAC2/3 voltage dependent-anion channel 2/3.

**Fig. 2:**
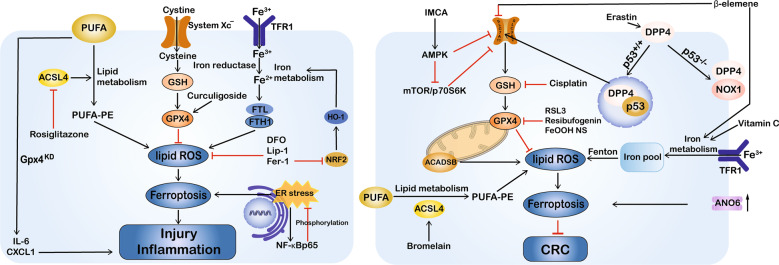
Dual regulatory roles for ferroptosis in intestinal diseases. Ferroptosis can be a positive and negative regulator of intestinal diseases according to cell type and disease context. The induction of ferroptosis by multiple compounds can inhibit cancer growth; however, inhibiting ferroptosis has an anti-inflammatory effect and can attenuate intestinal injury in IBD and I/R injury. This schematic diagram shows ferroptosis regulators and pathways. ACADSB acyl-Coenzyme A dehydrogenase short/branched chain, ACSL4 acyl-CoA synthetase long-chain family member 4, ANO6 anoctamin 6, CRC colorectal cancer, CXCL1 chemokine (C-X-C motif) ligand 1, DFO deferoxamine, DPP4 dipeptidyl-peptidase-4, ER endoplasmic reticulum, Fer-1 ferrostatin-1, FTL ferritin light chain, FTH1 ferritin heavy chain 1, FeOOH NS iron oxide-hydroxide nanospindle, GPX4 glutathione peroxidase 4, GSH glutathione, HO-1 haem oxygenase 1, IMCA 2-Imino-6-methoxy-2H-chromene-3-carbothioamide, I/R ischaemia/reperfusion, IBD inflammatory bowel disease, IL-6 interleukin 6, KD knockdown, Lip-1 liproxstatin-1, NOX1 NADPH oxidase 1, NRF2 nuclear factor E2-related factor 2, PUFA polyunsaturated fatty acid, PE phosphatidylethanolamine, ROS reactive oxygen species, RSL3 Ras-selective lethal small molecule 3, SLC7A11 solute carrier family 7 member 11, TFR1 transferrin receptor 1.

Ferroptosis is a form of regulated cell death (RCD) that was first proposed by Dixon and colleagues in 2012^1^. It is morphologically, biochemically, and genetically different from other kinds of RCD, such as apoptosis, necroptosis, and autophagy^1,2^. Iron metabolism and the lipid peroxidation pathway are central mediators of the ferroptotic process^3,4^ (Fig. 1). Excessive iron regulates ferroptosis by producing lethal reactive oxygen species (ROS) via the Fenton reaction, while reduced glutathione (GSH) depletion and/or glutathione peroxidase 4 (GPX4) inhibition trigger ferroptosis through the accumulation of intracellular lipid ROS and overwhelming lipid peroxidation^1,4,5^. In addition, ROS attack the polyunsaturated fatty acids (PUFAs) of lipid membranes, producing massive lipid peroxides and leading to membrane damage and cell death^4,6^. Specific small-molecule compounds, such as erastin and RAS-selective lethal 3 (RSL3) can induce ferroptosis, while ferrostatin-1 (Fer-1), liproxstatin-1 (Lip-1), and iron chelators deferoxamine (DFO) inhibit it^7,8^. Accumulating evidence suggests that ferroptosis participates in multiple diseases, including neurological disorders, ischaemia/reperfusion (I/R) injury, kidney failure, cardiac disease, and cancer^1,4,9–11^. Recent studies have also implicated ferroptosis in intestinal diseases, including intestinal I/R injury, inflammatory bowel disease (IBD), and colorectal cancer (CRC)^12–16^ (Fig. 2 and Table 1). Ferroptosis has been reported in ulcerative colitis (UC) in both humans and mice; moreover, blocking the ferroptotic process alleviated dextran sulphate sodium (DSS)-induced colitis^12,13^. Furthermore, ferroptosis can limit the migration, invasion, and proliferation of CRC. Indeed, RSL3 drives ferroptotic cell death by promoting cellular ROS accumulation and increasing iron load^17^. Another report indicated that in CRC, resibufogenin inhibited cell growth and tumorigenesis by inducing ferroptosis through GPX4 inactivation^18^. Taken together, ferroptosis appears to play a key role in the pathophysiological processes and may provide new ideas and means for the treatment of intestinal diseases. This review presents a comprehensive description of ferroptosis and its emerging role in multiple intestinal diseases.

**Table 1:** Mechanisms of ferroptosis in intestinal diseases.

Disease	Compound/target	Model	Effect	Mechanism	Ref.
Intestinal I/R injury	Lip-1	I/R mice; Caco-2 cells	Inhibition	Inhibition of ferroptosis ameliorated I/R-induced intestinal injury and ACSL4 could regulate ferroptosis-associated I/R injury.	^14^
Inflammatory bowel disease	Fer-1/ Lip-1/DFP	DSS-induced colitis mice	Inhibition	Ferroptosis mediated DSS-induced UC associated with NRF2/HO-1 signalling pathway.	^12^
	Fer-1/ DFO/GSK414	DSS-induced colitis mice; p65^IEC-KO^ mice; HCoEpiC cells	Inhibition	Ferroptosis contributes to UC via ER stress-mediated-IEC cell death and NF-κBp65 phosphorylation suppresses ER stress-mediated IEC ferroptosis to alleviate UC.	^13^
	Curculigoside	DSS-induced colitis mice; IEC-6 cells	Inhibition	Curculigoside inhibited ferroptosis in UC through the induction of GPX4.	^91^
Colorectal cancer	RSL3	HCT116/LoVo/ HT29 cells	Induction	RSL3 triggered ferroptosis via GPX4 inactivation and ROS production in CRC cells.	^17^
	Cisplatin	HCT116 cells	Induction	Cisplatin induced ferroptosis through GSH depletion and GPX4 inactivation.	^100^
	β-elemene	HCT116/Lovo cells; Orthotopic xenografts mice	Induction	Combinative treatment of cetuximab and β-elemene suppressed the growth and migration of KRAS-mutant CRC cells by triggering ferroptosis.	^99^
	Vitamin C	DiFi cells; CRC organoids	Induction	Vitamin C altered iron homoeostasis, increased ROS production and triggered ferroptosis.	^67^
	Resibufogenin	HT29/SW480 cells; Orthotopic xenografts mice	Induction	Resibufogenin induced ferroptotic cell death in a GPX4 inactivation-dependent manner.	^18^
	IMCA	DLD-1/HCT116 cells; Orthotopic xenografts mice	Induction	IMCA triggered ferroptotic cell death by downregulating SLC7A11 via the AMPK/mTOR signalling pathway in CRC.	^103^
	SLC7A11	HT29 cells	inhibition	Knockout of *SLC7A11* facilitated the ferroptotic cell death and kill colorectal cancer stem cells.	^105^
	Bromelain	HCT116/DLD1 cells; KRAS-mutant mice	Induction	Bromelain increased ROS-induced ferroptosis by increasing ACSL4 expression levels in KRAS-mutant CRC cells.	^106^
	p53	HCT116/SW48 cells; Tumour-bearing mice	Inhibition	p53 limited erastin-induced ferroptosis by blocking DPP4 activity in a transcription-independent manner	^61^
	MiRNAs (let-7c, let-7e, miR-150-5p)	CRC patient samples	Inhibition	Downregulated miRNAs including let-7c, let-7e and miR-150-5p modulated the *TP53* gene targeting the process of ferroptosis in CRC.	^107^
	Erastin/Artesunate	HCT116 cells; Orthotopic xenografts mice	Induction	The p53-independent PUMA axis is involved in ferroptosis in human colon cancer HCT116 cells.	^108^
	Sorafenib	HCT116/CX-1/LS174T cells; Orthotopic xenografts mice	Induction	Ferroptosis‐inducing agents, such as sorafenib enhanced TRAIL‐induced apoptosis through upregulation of DR5.	^109^
	NCOA4	HCT116/SW480 cells	NA	NCOA4 was not essential for ferroptosis in CRC cells.	^110^
	FeOOH nanospindles	CT26 cells	Induction	FeOOH nanospindles could induce ferroptosis by effectively scavenging endogenous hydrogen sulphide.	^111^
	ACADSB	SW620 cells	Induction	ACADSB mediated ferroptosis by negatively regulating expression of glutathione reductase and GPX4	^112^
	TMEM16F	TMEM16F KO mice HT29 cells	Induction	TMEM16F is activated during erastin and RSL3-induced ferroptosis, providing a finding that may be useful to induce cell death in CRC.	^113^

*ACADSB* acyl-Coenzyme A dehydrogenase short/branched chain, *ACSL4* acyl-CoA synthetase long-chain family member 4, *CRC* colorectal cancer, *DFP* deferiprone, *DFO* deferoxamine, *DPP4* dipeptidyl-peptidase-4, *DR5* death receptor 5, *DSS* dextran sulphate sodium, *ER* endoplasmic reticulum, *Fer-1* ferrostatin-1, *GPX4* glutathione peroxidase 4, *GSH* glutathione, *HO-1* haem oxygenase 1, *IEC* intestinal epithelial cell, *IMCA* 2-Imino-6-methoxy-2H-chromene-3-carbothioamide, *I/R* injury ischaemia/reperfusion injury, *KO* knockout, *Lip-1* liproxstatin-1, *NA* not applicable, *NCOA4* nuclear receptor coactivator 4, *NRF2* nuclear factor E2-related factor 2, *PUMA* p53 upregulated modulator of apoptosis, *ROS* reactive oxygen species, *UC* ulcerative colitis, *RSL3* Ras-selective lethal small molecule 3, *SLC7A11* solute carrier family 7 member 11, *TP53* tumour protein 53, *TRAIL* tumour necrosis factor-related apoptosis-inducing ligand.

## Ferroptosis: an iron-dependent type of regulated cell death with clinical significance

### Definition and measurement

Since 2003, Stockwell and colleagues have successively identified novel compounds, including erastin and RSL3, that activate new, nonapoptotic cell death in particular cancer cells^19,20^. Inhibitors specific to known RCDs did not inhibit this chemically induced cell death; however, antioxidants and iron chelators could block and reverse the process^21^. The definition of ferroptosis was proposed in 2012: nonapoptotic, iron-dependent cell death characterised by the accumulation of lipid peroxidation products and the depletion of membrane PUFAs^1^. Ferroptosis was added to the RCD family by the Nomenclature Committee on Cell Death (NCCD) in 2018^22^. Morphologically, ferroptosis manifests as small mitochondria with concentrated membrane density, decreased or vanishing mitochondrial cristae, and outer mitochondrial membrane rupture^4,23^. The biochemical properties of ferroptosis are iron accumulation, lethal ROS production, and overwhelming lipid peroxidation^4,10^. Multiple molecules, including GPX4, p53, solute carrier family 7 member 11 (SLC7A11), acyl-CoA synthetase long-chain family member 4 (ACSL4), NADPH oxidase (NOX), and nuclear factor E2-related factor 2 (NRF2) positively or negatively regulate ferroptosis^1,24–26^.

To assess ferroptosis, the Cell Counting Kit-8 and propidium iodide staining can be used to measure cell viability and death^9,27^. Measuring lipid peroxidation is important for evaluating the presence of ferroptosis in specific contexts. Oxidative lipidomics is the gold standard for identifying specific oxidized lipids^28,29^. Probes such as C11-BODIPY and Liperfluo provide indirect but efficient means to detect lipid ROS^2,30^. Moreover, malondialdehyde (MDA) and 4-hydroxynonenal (4-HNE) are common by-products of lipid peroxidation during oxidative stress that allow the measurement of lipid peroxidation^31^. Another method for evaluating ferroptosis is to test cellular iron levels using an iron assay kit or the fluorescent probe Phen Green SK (PGSK)^20,32^. We can also detect changes in ferroptosis-related gene expression, such as prostaglandin-endoperoxide synthase (*PTGS*), *ACSL4*, *GPX4*, and ferritin heavy chain 1 (*FTH1*)^33^. In addition, transmission electron microscopy can be used to identify specific morphological features of cells to support ferroptosis occurrence^34^.

### Mechanisms and mediators of ferroptosis

#### GSH/GPX4-lipid peroxidation pathway

Ferroptosis is triggered by excessive lipid peroxidation arising from iron-dependent ROS accumulation. As it can occur when GSH-dependent lipid peroxide repair systems are compromised, maintaining ROS and lipid peroxides at physiological concentrations is a critical factor in minimizing susceptibility^2,35^. Lipophilic antioxidants (e.g. Fer-1, Lip-1) have been defined as specific ferroptosis suppressors that inhibit ROS accumulation caused during lipid oxidation. GSH is a thiol-containing tripeptide that plays an essential role in intracellular antioxidant defences. Its depletion causes increased oxidative stress, macromolecular damage, and subsequent cell death^36^. GPX4 is a member of the glutathione peroxidase family capable of reducing cytotoxic lipid hydroperoxides (L-OOH) to non-toxic lipid alcohols (L-OH) or catalysing free hydrogen peroxide into water to prevent the formation and accumulation of lethal lipid ROS at the expense of GSH^37,38^.

Accumulating evidence has implicated GPX4 as a master regulator of ferroptosis; its inhibition by pharmacological or genetic methods can trigger ferroptotic cell death through the accumulation of lipid peroxides^5,39,40^. Indeed, RSL3 has been shown to induce ferroptosis by directly inhibiting GPX4 activity through covalent binding with the selenocysteine active site of GPX4^5,41^. GPX4 can also be inactivated by indirect methods, such as cellular GSH depletion. The biosynthesis of GSH requires the participation of glutamate, cysteine, and glycine in a two-step reaction catalysed by glutamate-cysteine ligase (GCL) and glutathione synthetase (GSS)^42,43^. Thus, GSH depletion can result either from direct inhibition of GSH synthesis (e.g. by the known GCL inhibitor buthionine sulphoximine (BSO)^6^) or from cysteine/glutamate unavailability. Cysteine, the rate-limiting substrate for GSH biosynthesis, is produced from dipeptide cystine imported by the cell surface cystine/glutamate antiporter system X_c_^−^, or from methionine via the transsulphuration pathway^44,45^. Inhibiting system X_c_^−^ can reduce GSH levels and GPX4 activity, contributing to ferroptotic cell death. Erastin and other molecules (e.g. sulphasalazine, sorafenib) are inhibitors of system X_c_^−^ and thus induce ferroptosis^1,3,4^. Studies have shown that regulating the expression of *SLC7A11*, the functional subunit of system X_c_^−^, affects system X_c_^−^ activity and ferroptosis sensitivity in cancer cells^24,26,44^. Furthermore, cysteinyl-tRNA synthetase (CARS) has been found to participate in the transsulphuration pathway; its knockdown causes upregulation of this pathway and erastin-induced ferroptosis resistance^2^.

As described below, disruption of lipid repair systems involving GSH and GPX4 can facilitate the accumulation of (lipid) ROS; however, cysteine/GSH depletion and/or GPX4 suppression alone is not sufficient to cause ferroptosis. ROS react with PUFAs of lipid membranes to cause lipid peroxidation, which is central to the final execution of ferroptosis. Free PUFAs are substrates for the synthetic-lipid signal-transduction medium, but they must be esterified and incorporated into membrane phospholipids (PLs) with the help of the enzymes ACSL4 and lysophosphatidylcholine acyltransferase 3 (LPCAT3). Then lipoxygenases (LOXs) catalyse PUFA‐containing PLs to produce pro‐ferroptotic lipid peroxidation^23^. Researchers have identified ACSL4 as both a biomarker for, and a critical contributor to, ferroptosis^25,46^. ACSL4 expression is positively correlated with ferroptosis sensitivity; in addition, lipid oxidation upon GPX4 inhibition requires the involvement of ACSL4^25,46^. One group has reported that *LPCAT3* deletion protected fibroblasts against ferroptosis, suggesting that LPCAT3 is also an important player in ferroptosis^25^. However, this protective effect was mild compared with the protection provided by ACSL4 deletion, suggesting that ACSL4 plays a more extensive role in ferroptosis; moreover, the functional effect of LPCAT3 possibly depends on cellular subtypes^25,47^. Of the different oxidised PL species, PUFA-containing phosphatidylethanolamines (PEs), especially arachidonic acid (AA)- and adrenic acid (AdA)-containing PEs, are the most susceptible to peroxidation in ferroptosis^48^. Finally, overwhelming lipid peroxidation likely alters lipid bilayer properties, producing cytotoxic reactive fragments, and leading to irreversible cell death^49^.

#### Iron metabolism and ferroptosis

Iron is a redox-active element that promotes ROS generation through the Fenton reaction, which leads to non-enzymatic lipid peroxidation^3,50^. Iron also serves as a cofactor for iron-containing enzymes, including LOXs, suggesting a necessary role in enzymatic lipid reactions^50^. Thus, as a significant factor for the production of (lipid) ROS via enzymatic or non-enzymatic ways, iron appears to be an indispensable component in ferroptosis^1,2^. The chelation of intracellular iron by DFO and ciclopirox olamine is sufficient to inhibit erastin-induced cell death, reinforcing the importance of iron in ferroptosis^4^. Normally, extracellular iron forms a complex with circulating transferrin (TF), binds to the specific membrane transferrin receptor protein-1 (TFR1), and is delivered into cells. Excess cellular iron is stored as ferritin, the main intracellular iron storage protein that consists of a ferritin light chain (FTL) polymer and FTH1, or exported by iron exporter ferroportin (FPN)^51,52^. Maintaining cellular iron homoeostasis can prevent oxidative damage, and cell toxicity and death. Either reduced iron storage or increased iron uptake can cause iron overload and trigger ferroptosis^4^. Recent studies have revealed an association between genes involved in iron metabolism and ferroptosis. Erastin-induced ferroptosis can be prevented by silencing *TFRC*, the gene that encodes TFR1, whereas overexpression of haem oxygenase 1 (*HO-1*) alters iron homoeostasis and aggravates it^6,53^. Autophagic degradation of ferritin, known as ferritinophagy, modulated by the nuclear receptor coactivator 4 (NCOA4), controls cellular liable iron levels and ROS accumulation, thus regulating ferroptotic cell death in some cell lines^54,55^. The pentaspan membrane protein prominin-2 can drive ferroptosis resistance by promoting the formation of ferritin-containing multivesicular bodies and exosomes, thus exporting iron from the cell^56^. Furthermore, iron response element-binding protein 2 (*IREB2*) encodes the master regulator of iron metabolism, which results in the expression of *TRFC*, *FTH1*, and *FTL*. Inhibiting *IREB2* expression contributes to decreased sensitivity to erastin-induced ferroptosis^1^. Other targets, such as heat shock protein beta-1 (HSPB1) and CDGSH iron sulphur domain 1 (CISD1), can also regulate ferroptotic cell death by mediating iron uptake and lipid peroxidation^57,58^. Collectively, these findings indicate the iron dependence of ferroptosis.

#### Other ferroptosis regulatory pathways

**Fig. 3:**
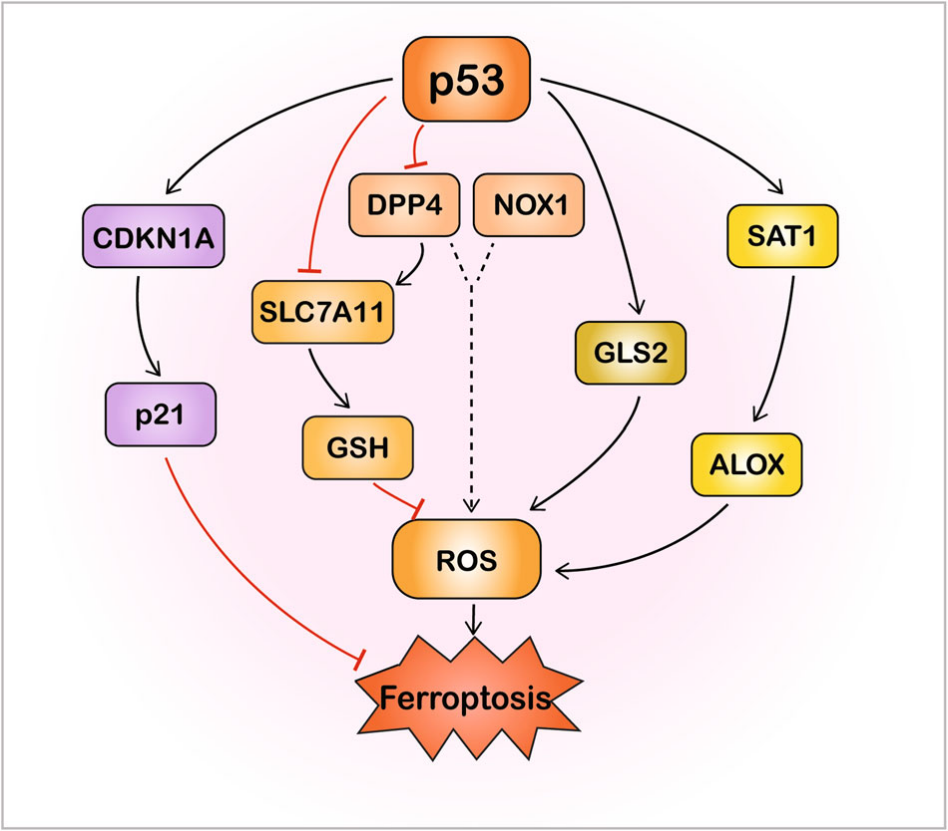
p53-mediated ferroptosis. p53 plays a dual role in the regulation of ferroptosis through transcriptional or posttranslational mechanisms in different contexts. On one hand, p53 induces ferroptosis through inhibition of SLC7A11 or upregulation of GSL2 and SAT1–ALOX pathway. On the other hand, p53 also inhibits ferroptosis through inhibition of DPP4 activity or by the transcriptional activation of CDKN1A/p21. ALOX arachidonate lipoxygenase, CDKN1A cyclin-dependent kinase inhibitor 1 A, DPP4 dipeptidyl-peptidase-4, GLS2 glutaminase 2, GSH glutathione, NOX1 NADPH oxidase 1, ROS reactive oxygen species, SAT1 spermidine/spermine N1-acetyltransferase 1, SLC7A11 solute carrier family 7 member 11.

The canonical tumour suppressor p53 probably plays dual roles in mediating ferroptosis in multiple cancers (Fig. 3). It can directly adjust the metabolic versatility of cells by modulating metabolic targets, favouring mitochondrial respiration and resulting in ROS-mediated cell death^59^. Researchers have found that p53 represses SLC7A11 protein expression, resulting in decreased cystine import, decreased GSH production, and enhanced ROS-mediated ferroptosis in some cancer cell lines^24,59^. The acetylation-defective mutant p53^3KR^, which lacks the ability to trigger apoptosis, cell-cycle arrest, and senescence, can suppress tumourigenesis by inhibiting SLC7A11 and inducing ferroptosis^24^. On the contrary, other studies have reported an inhibitory effect of p53 on ferroptosis in different contexts. Wild-type p53 stabilisation suppresses ferroptosis in specific cancer cell lines in response to cystine deprivation and system X_c_^−^ inhibition because of the activation of p53–p21 transcriptional axis^60^. Besides, p53 negatively regulates ferroptosis in CRC cells by inhibiting dipeptidyl-peptidase-4, described in more detail in section ‘Ferroptosis and colorectal cancer’ below^61^. In addition to p53-mediated ferroptosis, the intracellular metabolic process glutaminolysis is required for the initiation of cystine deprivation-induced ferroptosis^9^. The FSP1–CoQ10–NAD(P)H pathway exists as an independent parallel system that cooperates with GSH/GPX4 to mitigate lipid peroxidation and ferroptosis^62^. Targeting the NRF2-related pathway is also a vital strategy to mediate lipid peroxidation and ferroptosis^63^.

### The significance of ferroptosis research in disease

In parallel with more basic research, it has been found that inducing or blocking ferroptosis can affect the onset and development of multiple pathogenic conditions, providing a potential target for therapeutics, especially for diseases tolerant/resistant to conventional drugs. Taking drug-resistant cancer as an example, ineffective induction of cancer cell death is an important problem with many chemotherapy and bio-targeted drugs, closely linked to drug resistance^64^. Persister cells are clones that survive initial cancer treatment and induce drug-resistant states across diverse cancer contexts^64,65^. Interestingly, induction of ferroptosis can kill these drug-tolerant persister cells and decrease the emergence of acquired drug resistance^66,67^. In addition, the epithelial-to-mesenchymal transition (EMT) is one of the mechanisms leading to apoptotic failure and drug resistance in epithelial-derived carcinoma cells^68^. Evidence has indicated that tumour cells in a high-mesenchymal state are characterised by enhanced activity of enzymes related to the promotion of PUFA synthesis, making these cells dependent on the lipid peroxidase pathway involving GPX4. Thus, cancer cells in a mesenchymal state can undergo ferroptosis through pharmacological perturbations to overcome cancer therapy resistance^68^. Targeting ferroptosis is a new perspective for the treatment of kidney injury, non-cancer liver diseases, and intestinal diseases, suggesting the significant potential of ferroptosis research^10,69^.

## Ferroptosis in intestinal disease

### Ferroptosis and intestinal ischaemia/reperfusion (I/R) injury

Intestinal I/R injury is a common clinical condition with high morbidity and mortality, resulting from sudden reduction of intestinal blood flow and reoxygenation after the restoration of blood supply^70^. It occurs in many clinical conditions, including trauma, haemorrhagic shock, acute mesenteric ischaemia, small intestinal volvulus, and intestinal transplantation^71,72^. Intestinal mucosal barrier dysfunction, as a consequence of epithelial cell death, can allow the translocation of bacteria and associated toxins into the bloodstream, leading to inflammation, systemic sepsis, and organ dysfunction^73,74^. Intestinal I/R injury is associated with multiple types of RCD, including apoptosis, necroptosis, and autophagy, but with the discovery of ferroptosis, new potential mechanisms of RCD have attracted attention^75–77^. Indeed, ferroptosis has been identified in I/R injury in other organs both in vivo and in vitro; moreover, ferroptosis inhibitors can alleviate these injuries. Studies have shown that treatment with DFO reduced myocardial infarct size and lactate dehydrogenase levels in an ex vivo heart model of I/R stress^9^, while Lip-1 prevented acute renal failure from renal I/R injury^39^. ROS generation and lipid peroxidation are associated with intestinal I/R injury and are primary contributors to the initiation and execution of ferroptosis^78^. Decreased GSH levels and superoxide dismutase activity, as well as increased MDA levels, were observed in rat intestinal tissues after intestinal I/R^79,80^. Furthermore, DFO administration was beneficial in the prevention of intestinal I/R-induced lipid peroxidation and GPX activity reduction was reversed by DFO treatment^81^. Taken together, lipid peroxidation and iron participate in I/R-induced intestinal injury, but their contribution to ferroptosis is still enigmatic.

A recent study has reported that ferroptosis plays a critical role in intestinal I/R injury and may be a lethal process triggered by reperfusion^14^. In this study, the expression levels of pro-ferroptotic factors such as ACSL4 and iron increased, while those of anti-ferroptotic factors (GPX4, FTH1, GSH) decreased in ischaemic murine intestinal tissues; moreover, treatment with Lip-1 ameliorated intestinal injury both in vivo and in vitro^14^. Moreover, an ischaemia model that incorporated different reperfusion durations to examine features of ferroptosis in situ suggested that this form of cell death occurred in the early phase of reperfusion and was distinct from apoptosis, which appeared at a later phase^14^. Inhibition of ischaemia-induced ACSL4 (a key regulator and indicator of ferroptosis) expression via pharmacological and genetic manipulations protected against lipid peroxidation and ferroptosis, as well as alleviated cell damage and intestinal barrier dysfunction induced by intestinal I/R^14,25^. Li and colleagues have further shown that Sp1, a transcription factor that binds to GC-box motifs in target-gene promoters, mediated ACSL4 expression^14^. In addition to intestinal damage, intestinal I/R can cause acute injury to remote organs, including the lungs and liver. Indeed, ferroptosis was reported to exacerbate intestinal I/R-induced acute lung injury, whereas blocking this process mitigated lung injury after intestinal I/R in mouse models^14,82^. In summary, ferroptosis is related to I/R-induced intestinal injury, but more studies are needed to discover its underlying mechanisms and regulation.

### Ferroptosis and inflammatory bowel disease

Inflammatory bowel disease (IBD), including ulcerative colitis (UC) and Crohn’s disease (CD), is a chronic disease characterised by constant progression and relapse. Although not fully elucidated, the aetiology of IBD is commonly thought to implicate reciprocal interactions between host genetics, environmental factors, the gut microbiome, and immune responses^83^. A better understanding of IBD pathogenesis will be beneficial for improving its treatment; recent studies have underlined the importance of cell death in intestinal epithelial homoeostasis. Excessive cell death is closely correlated with chronic inflammation in IBD patients^84^, but what is the relationship between ferroptosis and IBD? It has been reported that iron supplementation changes gut microbial homoeostasis and exacerbates intestinal inflammation similarly to CD in a murine model^85^. Another study using a rat model of DSS-induced colitis also indicated that excess iron aggravated intestinal inflammation^86^. Recently, a Japanese team showed that high dietary iron intake increased the risk of UC^87^. Mucosal ROS production is increased in UC in proportion to the disease activity, and iron chelators are known to reduce ROS production and ameliorate colonic symptoms in IBD^88,89^. Taken together, these findings suggest a possible relationship between IBD and ferroptosis in which excess iron in the intestine produces ROS via the Fenton reaction, which triggers oxidative stress. Lipid peroxidation procedurally appears and ferroptotic cell death is induced. Thereby, the intestinal epithelial cells are destroyed, and damage to the intestinal mucosal barrier results in IBD^90^.

Ferroptosis has been implicated in both clinical UC patients and in murine experimental colitis, with significant downregulation and upregulation of ferroptosis-associated genes^12,13^. Administration of ferroptosis inhibitors, including Fer-1, Lip-1, and DFO, reduced disease activity scores and ameliorated colon length shortening in DSS-induced murine colitis, suggesting the beneficial effect of inhibiting ferroptosis^12,13,91^. In keeping with ferroptosis mechanisms, GPX4 also plays a vital role in negatively regulating ferroptotic cell death in IBD. Curculigoside (CUR) is a botanical ingredient with anti-oxidant and anti-inflammatory properties that protects against ferroptosis in UC through the promotion of GPX4^91^. CUR increased selenium sensitivity and enhance GPX4 expression levels in the IEC-6 rat intestinal epithelial cell line, while *Gpx4* silencing inhibited the protective effects of CUR on cell death and oxidative stress indicators in ferroptotic IEC-6 cells^91^. Moreover, another group emphasised the importance of GPX4 in gut homoeostasis by showing impaired GPX4 activity and features of lipid peroxidation in the small intestinal epithelial cells (IECs) of CD patients^92^. PUFAs, especially AA, induced the production of interleukin 6 (IL-6) and chemokine (C-X-C motif) ligand 1 (CXCL1) in IECs treated with *Gpx4* siRNA in response to iron availability, lipid peroxidation, and ACSL4, similar to ferroptosis mechanisms^92^. Interestingly, a PUFA-enriched Western diet triggered small intestinal inflammation in mice that lacked one *Gpx4* allele in IECs, with histological characteristics resembling CD^92^. The link between PUFA uptake, GPX4 activity, and intestinal inflammation further provides evidence for the pathogenesis of CD. However, although the process observed in the study was similar to ferroptosis, no definite cell death was observed in the murine intestinal inflammation model; the scholars speculate that in this case, one *Gpx4* allele might be sufficient to protect against ferroptotic cell death^92^.

NRF2 is a critical mediator of the cellular antioxidant response that controls redox homoeostasis-related gene expression; perturbations of the NRF2-lipid peroxidation–ferroptosis axis have been found in cancers^63^. HO-1, a cytoprotective enzyme related to cellular stress, also participates in ferroptosis and has anti-inflammatory effects^93^. Chen et al. found that Fer-1 alleviated DSS-induced colitis via NRF2/HO-1 signalling, indicating that the NRF2 pathway may be an important factor regulating ferroptosis in UC^12^. ER stress can induce the cell-death signalling pathway in the form of apoptosis and autophagy^94,95^, but interestingly, ER stress also is implicated in the development of ferroptosis in diseases, including IBD^13,96^. It has been found that ferroptosis contributed to UC via ER stress-mediated IEC cell death; moreover, phosphorylation of NF-κBp65 inhibited ER stress-mediated IEC ferroptosis to relieve the disease as an upstream regulator^13^. Together, these data show that ferroptosis has a key role in IBD, and that targeting it may be a promising method for understanding the development of IBD and to provide new treatments.

### Ferroptosis and colorectal cancer

Colorectal cancer (CRC) is a common malignant tumour with high morbidity and mortality and is one of the most pressing global health issues. According to the GLOBOCAN 2018 estimates of incidence and mortality worldwide report, CRC is the third-most diagnosed cancer and the second-most cause of cancer-related deaths globally^97^. Current treatments for CRC include surgery, radiotherapy, chemotherapy, immune therapy, and bio-targeted therapy^98^; however, despite recent progress in therapeutics, some patients exhibit resistance or intolerance to them via apoptosis evasion and anti-apoptotic enhancement^99,100^. Thus ferroptosis, as a form of RCD independent from apoptosis, may provide a promising strategy for cancer therapy. Since its discovery in 2012, the manipulation of ferroptosis by specific molecules has enabled inhibition of the growth and spread of multiple cancer types, including CRC^1,23^. RSL3-induced ferroptosis in a dose-and time-dependent manner in three CRC cell lines; this treatment increased ROS and cellular labile iron pool (LIP) levels^17^. Evidence has showed that the classic chemotherapy drug cisplatin induces ferroptosis; moreover, the combination of cisplatin and erastin was synergistic, indicating that ferroptosis adds an alternative cell-death pathway triggered by classical therapeutic drugs and anti-tumour mechanisms in CRC^100^. In addition, targeting ferroptosis can overcome conventional CRC drug resistance from a new perspective. Chen et al. reported that the bioactive compound β-elemene (extracted from the Chinese herb Curcumae Rhizoma) is a ferroptosis inducer; they combined treatment with β-elemene and anti-EGFR (epidermal growth factor receptor) antibody cetuximab to produce anti-tumour effects by triggering ferroptosis in CRC patients with *RAS* mutations that do not respond to cetuximab^99,101^. Another study showed that vitamin C, an antioxidant that can paradoxically initiate oxidative stress at pharmacological doses, targeted cetuximab-persister cells and restricted the emergence of acquired resistance to EGFR blockade in CRC through the induction of ferroptosis^67^. Altogether, the role of ferroptosis in CRC in inhibiting tumour growth and overcoming resistance to current anticancer drugs is a promising avenue for research.

As described above, GPX4 plays a central role in regulating ferroptosis. Recently, several molecules have been implicated in ferroptosis in CRC through their mediation of GPX4. RSL3 inhibits GPX4 activity by directly binding with GPX4, and overexpression of GPX4 rescued CRC cell death induced by RSL3, suggesting a similar role of GPX4 in RSL3-induced ferroptosis in CRC as in other diseases^17^. Shen et al. found that resibufogenin isolated from Asiatic toad dried skin secretions is a potential anticancer agent in the treatment of CRC because it induced ferroptosis in a GPX4 inactivation-dependent manner^18^. In addition to direct GPX4 inhibition, inhibiting SLC7A11, the functional subunit of system X_c_^−^, also induces ferroptotic cell death in CRC. It was reported that the benzopyran derivative 2-imino-6-methoxy-2H-chromene-3-carbothioamide (IMCA) has a wide spectrum of biological activities, including those relevant to cancer therapy^102^. Zhang et al. first discovered the anti-CRC effect of IMCA through ferroptosis induction by downregulating SLC7A11^103^. Interestingly, IMCA affected the downstream components of the AMPK/mTOR/p70S6k pathway, which have been linked to SLC7A11 activity and ferroptosis^103^. The role of SLC7A11 and ferroptosis has also been elucidated in colorectal cancer stem cells (CSCs), which can provide resistance to chemotherapy and form secondary tumours in the progression of CRC^104^. Colorectal CSCs are more sensitive to ferroptosis than parental CRC cells; the knockout of *SLC7A11* with CRISPR-Cas9 technology facilitated ferroptotic cell death, suggesting that targeting SLC7A11 may specifically suppress the progression of colorectal CSCs and reduce CRC drug resistance^105^. Another key regulator of ferroptosis in many related diseases is ACSL4^25^. A recent study has determined its crucial regulatory role in the induction of ferroptosis by bromelain (a plant extract derived from pineapple) in *KRAS*-mutant CRC through signalling pathway and miRNA profiling^106^.

The *TP53* gene is known as a tumour suppressor and ferroptosis regulator in multiple cancers. It inhibited ferroptotic CRC cell death by blocking dipeptidyl-peptidase-4 (DPP4) activity, while the loss of p53 increased the anticancer activity of erastin in tumour-bearing mice, very different from the positive regulation of ferroptosis by p53 in other cancers (Fig. 3)^61^. Specifically, the loss of p53 restrains DPP4 nuclear localisation and facilitates the formation of the DPP4–NOX1 complex that promotes lipid peroxidation, resulting in ferroptosis in the HCT116 human CRC cell line^59,61^. While p53 can limit ferroptosis by forming a DPP4–p53 complex in the nucleus, disassembly of this complex restores the erastin-induced ferroptosis sensitivity of CRC^59,61^. Moreover, the fact that TP53 can stimulate SLC7A11 expression in CRC protects CRC cells from ferroptosis^61^. Therefore, regulation of TP53 may be highly desirable as part of CRC therapy. In a human miRNome analysis of miRNA–mRNA interactions and multiple pathways involved in CRC pathogenesis, three downregulated miRNAs, let-7c, let-7e, and miR-150-5p, were found to modulate *TP53* in CRC and thus could regulate ferroptosis^107^.

There also are connections between ferroptosis and other types of RCDs in CRC. Hong et al. reported molecular crosstalk between ferroptosis and apoptosis when CRC cells were treated with the ferroptotic agents erastin or artesunate (ART) in combination with the apoptotic agent tumour necrosis factor-related apoptosis-inducing ligand (TRAIL)^108^. The combination of erastin/ART and TRAIL significantly promoted TRAIL-induced apoptosis due to ER stress-induced p53-independent PUMA (p53 upregulated modulator of apoptosis) expression^108^. The group further found that ER stress response-mediated expression of the TRAIL receptor death receptor 5 (DR5) also played a vital role in this combinatorial synergy in a variety of CRC cell lines^109^. These studies are preliminary explorations of the mechanisms that may be shared between ferroptosis and apoptosis, but further research is needed to understand the relationship between these RCDs. Autophagy promotes ferroptotic cell death through the degradation of ferritin (ferritinophagy) in fibroblasts and certain cancer cells, modulated by selective cargo receptor NCOA4^54,55^; its inhibition suppresses ferritin degradation and inhibits ferroptosis in these cells^54,55^. However, this is not the case for CRC cells; Hasan et al. indicated that ferritinophagy was not required for CRC cell growth^110^. Interestingly, knocking out *NCOA4* did not alter ferroptosis in CRC^110^. Differences in cell lines may partially explain these conflicting findings, but another possibility is that CRC cells have an alternative mechanism that compensates for the loss of NCOA4 function in response to ferroptosis induction. Future studies should investigate the compensatory and alternative pathways in CRC that enhance cell survival.

Taken together, ferroptosis plays a significant role in CRC, and its regulation may provide new insights into cancer therapy. In addition to the regulators already mentioned, researchers also have identified novel compounds, for example, iron oxide-hydroxide nanospindles, with the potential to promote ferroptosis to inhibit colon cancer^111^. Researchers also have identified new targets which can regulate ferroptosis in CRC, including the short/branched chain acyl-coenzyme A dehydrogenase and anoctamin 6^112,113^. We believe that fully understanding ferroptosis and its underlying mechanisms in CRC, as well as the connections between ferroptosis and other RCDs, can give us hope to improve the treatment and prognosis of this cancer.

## Conclusions and perspectives

Ferroptosis is a newly identified type of RCD that is mediated by the iron-dependent accumulation of lipid ROS and has been implicated in the development of a wide variety of disorders, especially intestinal diseases. Inhibiting ferroptosis can attenuate intestinal injury in non-infectious inflammatory conditions such as intestinal I/R injury and IBD, while inducing ferroptosis with pharmacological activators can inhibit the migration, invasion, and proliferation of colorectal neoplasms, suggesting a dual role for ferroptosis in different intestinal diseases. As shown in Fig. 2, the common ferroptotic mechanisms in intestinal diseases include GPX4 inhibition, system X_c_^−^ suppression, lipid peroxide accretion, and iron overload, which are consistent with other diseases. Key regulators such as GPX4, SLC7A11, ACSL4, and p53 are also important for mediating ferroptosis-associated intestinal diseases. In addition to known ferroptotic inducers (erastin, RSL3) and inhibitors (Fer-1, Lip-1, DFO), researchers have found more drugs and targets related to ferroptosis in intestinal diseases (Table 1). However, whether there are specific regulators or signalling pathways in these diseases remains unclear. Interestingly, some regulators seem to play different roles in intestinal diseases compared with diseases in other organs, for example, p53 and NCOA4 in CRC. As a result, further research is needed to identify disease-specific ferroptotic mechanisms to develop disease context-dependent therapeutic regimens. Furthermore, studies have found correlations between ferroptosis and other forms of cell death in intestinal diseases. These RCDs may share common pathways and key regulators, which can provide new directions for combining different therapeutic interventions.

Although much progress has been made, research on intestinal ferroptosis is still at an early stage, and its specific role remains to be investigated across the spectrum of intestinal disease, including many not presented in this review. Although we have summarized multiple methods for assaying ferroptosis from multiple aspects, no unanimously agreed-upon criteria directly define its occurrence. It is imperative to identify markers and other approaches to assess ferroptosis in vivo. In this way, ferroptosis biomarkers could be promising to indicate intestinal disease severity. We caution that the relationship between ferroptotic cell death and iron/lipid peroxides remains controversial, so more evidence is required to support the links between ferroptosis and iron, oxidative stress, and lipid peroxidation in disease development and progression. In addition, the signalling pathways and main transcriptional regulators of ferroptosis need to be studied so that we can benefit more from its modulation to protect the intestine against injury and carcinogenesis. Therefore, ferroptosis should be further investigated within the field of intestinal disease as a novel therapeutic target.
